# Biomedical Imaging: Role and Opportunities of Medical Imaging in the “Omics” Era

**DOI:** 10.1155/2014/930213

**Published:** 2014-06-04

**Authors:** Tzu-Chen Yen, Dimitris Visvikis, Tinsu Pan, Yu-Hua Dean Fang

**Affiliations:** ^1^Center for Advanced Molecular Imaging and Translation, Chang Gung Memorial Hospital, Linkou 333, Taiwan; ^2^Department of Nuclear Medicine, Chang Gung Memorial Hospital, No. 5, Fu-Hsin Street, Kweishan, Linkou, Taoyuan 333, Taiwan; ^3^INSERM, UMR1101 LaTIM, CHRU Brest, 29609 Brest, France; ^4^Division of Diagnostic Imaging, Department of Imaging Physics, The University of Texas MD Anderson Cancer Center, Houston, TX 77030, USA; ^5^Department of Electrical Engineering, Chang Gung University, Taoyuan 333, Taiwan

In recent decades, we have witnessed an explosive growth of diagnostic tools that have changed the face of modern medicine. Among these diagnostic tools, medical imaging is indeed one of the most representative fields that have been growing fast, used extensively, and regarded as an irreplaceable part of clinical routines. With a very different scale, “omics” refers to the study of cellular- or DNA-level molecules including their functions, structures, interactions, and involvement in disease development. Similar to imaging, the “omics” fields such as genomics, proteomics, and metabolomics have also advanced drastically in recent decades. The “omics” data obtained from a patient reveal a much more microscaled world that allows the practitioner to see what is going on in the genomic and proteomic levels. As imaging and omics technologies look at human physiology in quite different scales, physicians and scientists are currently attempting to integrate imaging and omics data for tailored therapies and research purposes. This special issue provides original research and review on the potential role and value of imaging in the context of personalized medicine based on omics information.

Imaging can be used as a tool to directly measure the omics data. As pointed out by the comprehensive review by G. Lin and Y. L. Chung on the use of molecular imaging methods to study metabolomics in this issue, recent technological advancements of parallel imaging and high-field magnets have largely propelled the advancement of metabolomics for MR spectroscopy to measure the amount and exchange of multiple metabolites. Such imaging technologies can also be applied to in vivo imaging for both small animals and humans. Imaging the living organisms allows investigators to observe the metabolism and molecular exchange in both the spatial and temporal domains. In addition to measuring the omics data with imaging, clinical oncology continues to use medical images for treatment planning. G. C. Pereira et al. have given a comprehensive review on the role and importance of imaging in radiation therapies in this special issue. In this review, the critical role of imaging in radiation therapy is reviewed for the application of dose calculation, lesion location, and delineation. The combination of omics information and the image-derived lesion information has a high potential to further enhance the treatment efficacy for oncological applications.

Image quantification and information extraction remain to be a challenge for integrating imaging and omics information. In this special issue, Y.-H. D. Fang et al. described an example in the open-source software development for quantifying the intratumoral heterogeneity. Such quantification based on texture analysis has been reported to be helpful for prognosis in oncological applications. However, currently there is no free software for such quantification in the public domain. Therefore, Y.-H. D. Fang et al. developed and shared a software package to fill this void and demonstrated its usefulness in a small cohort of oral cavity cancer patients.

There are also many practical limitations for medical imaging that is modality-dependent. Ionizing radiation is one major concern for CT and radionuclide imaging such as PET and SPECT. MR does not require ionizing radiation and owns a great potential in molecular imaging, but it has its own limitations in speed, cost, and potential renal toxicity with the use of Gd-based contrast agents. In this special issue, H. M. Huang and Y. Y. Shih gave a review on the recent technical advancement of dose reduction for CT and acceleration for MR. From their review, it could be optimistically expected that such practical limitations will gradually be resolved to make those two modalities more commonly used in the integration of imaging and omics research in the future.

To fully exploit the omics data in personalized medicine, new advancements for instrumentation to extract omics data play a critical role. A review article by C.-S. A. Gong and K. F. Lei in this special issue will discuss the recent advancement of miniaturized devices for genomics, which may further advance the clinical popularity for gene sequencing. One of the striking examples is the use of electrochemical impedance spectroscopy as the electrical detection of DNA hybridization in microfluidic devices. The combination of microfluidic and impedimetric technologies shows an alternative and attractive method for detecting the genomic signal.

In conclusion, the dramatic advancements of the omics knowledge have further propelled the development of personalized medicine. The original and review articles in this special issue show that there will be a significant role of medical images in the future of personalized medicine. It can be optimistically expected that imaging will continue to evolve as part of the omics data as well as serving as a tool for omics measurements. Although integration of imaging and omics data is a challenging task for the imaging community, such efforts will truly benefit personalized medicine by allowing us to see an individual from macro- to microscale levels.

*Tzu-Chen Yen* *Dimitris Visvikis* *Tinsu Pan* *Yu-Hua Dean Fang*

